# Lymphatic MAFB regulates vascular patterning during developmental and pathological lymphangiogenesis

**DOI:** 10.1007/s10456-020-09721-1

**Published:** 2020-04-19

**Authors:** Lothar C. Dieterich, Carlotta Tacconi, Franziska Menzi, Steven T. Proulx, Kübra Kapaklikaya, Michito Hamada, Satoru Takahashi, Michael Detmar

**Affiliations:** 1 Institute of Pharmaceutical Sciences, ETH Zurich, 8093 Zurich, Switzerland; 2 Department of Anatomy and Embryology, Faculty of Medicine, University of Tsukuba, Tsukuba, Ibaraki 305-8575 Japan; 3 ETH Zurich, HCI H303, Vladimir-Prelog-Weg 3, 8093 Zurich, Switzerland

**Keywords:** Lymphangiogenesis, Vascular morphogenesis, Branching, Transcription factor, Postnatal development

## Abstract

MAFB is a transcription factor involved in the terminal differentiation of several cell types, including macrophages and keratinocytes. MAFB is also expressed in lymphatic endothelial cells (LECs) and is upregulated by VEGF-C/VEGFR-3 signaling. Recent studies have revealed that MAFB regulates several genes involved in lymphatic differentiation and that global Mafb knockout mice show defects in patterning of lymphatic vessels during embryogenesis. However, it has remained unknown whether this effect is LEC-intrinsic and whether MAFB might also be involved in postnatal lymphangiogenesis. We established conditional, lymphatic-specific Mafb knockout mice and found comparable lymphatic patterning defects during embryogenesis as in the global MAFB knockout. Lymphatic MAFB deficiency resulted in increased lymphatic branching in the diaphragm at P7, but had no major effect on lymphatic patterning or function in healthy adult mice. By contrast, tumor-induced lymphangiogenesis was enhanced in mice lacking lymphatic MAFB. Together, these data reveal that LEC-expressed MAFB is involved in lymphatic vascular morphogenesis during embryonic and postnatal development as well as in pathological conditions. Therefore, MAFB could represent a target for therapeutic modulation of lymphangiogenesis.

**Electronic supplementary material:**

The online version of this article (10.1007/s10456-020-09721-1) contains supplementary material, which is available to authorized users.

## Introduction

V-maf musculoaponeurotic fibrosarcoma oncogene homolog B (Mafb) belongs to the Maf family of basic leucine zipper transcription factors (TFs) which comprises the “large” Mafs (c-Maf, Mafa, Mafb, and Nrl) and the “small” Mafs (Maff, Mafg, and Mafk) that are characterized by the presence or absence of a transactivation domain, and thus act as transcriptional activators or repressors [1]. Mafb forms both homodimers and heterodimers with other basic leucine zipper TFs, including other Mafs, c-Fos, and c-Jun [2] and regulates gene expression by directly binding to Maf recognition elements (MARE) in promoter and enhancer elements. It is expressed in multiple tissues and has been linked to the differentiation of diverse cell types, including macrophages [3], keratinocytes [4, 5], kidney podocytes [6], and pancreatic α-cells and β-cells [7, 8].

Recently, we and others have found that MAFB also plays an important role in the embryonic development of the lymphatic vascular system [9, 10]. In mice, this process is initiated at embryonic day (E) 9.5, when lymphatic endothelial cell (LEC) progenitors transdifferentiate from venous endothelial cells in the cardinal vein, acquiring expression of the LEC “master regulator” TF Prox1 [11] via coordinated activity of two other TFs, SOX18 and NR2F2 [12, 13]. Prox1-expressing endothelial cells bud off the vein between E10.5 and E11.5 and migrate into the periphery to establish the primordial lymph sacs, which subsequently give rise to a primitive lymphatic plexus that further differentiates into the mature lymphatic system comprising lymphatic capillaries, collectors, and secondary lymphoid organs (reviewed in [14]). In addition, a non-venous origin of a subset of dermal, mesenteric, and cardiac LECs has been described [15, 16].

Signaling by vascular endothelial growth factor (VEGF) C/VEGF receptor (VEGFR) 3 is essential for lymphatic development as it controls the budding/migration process of LEC progenitors [17] from the vein, but also maintains the differentiated LEC phenotype [18]. However, the precise transcriptional events downstream of VEGFR-3 activation are still not fully understood. We have recently identified TFs activated by VEGFR-3 signaling in LECs [10, 19, 20] and found that MAFB is rapidly but transiently induced upon receptor stimulation and that it regulates expression of several important LEC differentiation and marker genes, including Prox1, Klf4, Flt4 (VEGFR-3), LYVE-1, and podoplanin [10]. Furthermore, we found that global Mafb deficiency resulted in abnormal patterning of developing lymphatic vessels in the back skin of mice at embryonic day E14.5, characterized by hyper-branching without an obvious decrease in overall lymphatic vessel growth [10]. Similarly, Koltowska et al. showed that the homologue gene *mafba* is important for normal lymphatic development in zebrafish and that its expression in LECs depends upon SOX18 and VEGF-C stimulation [9].

Since Mafb is highly expressed in various cell types in the developing mouse, including macrophages that are important regulators of lymphatic development [21], it has remained unclear whether the lymphatic phenotype observed during embryonic lymphatic development in global Mafb knockout mice was due to LEC-intrinsic effects or to indirect effects via macrophages. Furthermore, global Mafb knockout mice die perinatally due to a breathing defect [22], thus prohibiting studies of the postnatal function of MAFB in lymphangiogenesis. Lymphatic development does not stop at birth, but continues throughout the growth phase of the organism. For example, the lymphatic network in the mouse diaphragm continues to expand and remodel at least until postnatal day (P) 7 [23]. Additionally, de novo lymphangiogenesis can be induced in adult mice in pathological conditions, such as inflammation [24] and tumor growth [25, 26]. Lymphangiogenesis appears to play a beneficial role in acute and chronic inflammatory conditions, reducing edema and supporting the re-establishment of tissue homeostasis [27–29], whereas in many tumor types, lymphangiogenesis correlates with lymphatic metastasis and a poor outcome [25, 26]. Thus, a better understanding of how pathological lymphangiogenesis is regulated may open up new opportunities for therapeutic modulation of this process.

Here, we used both in vitro and in vivo approaches to investigate the LEC-intrinsic function of MAFB during embryogenesis, postnatal development, and pathological lymphangiogenesis.

## Results

### LEC-expressed MAFB regulates tubular morphogenesis in vitro

**Fig. 1:**
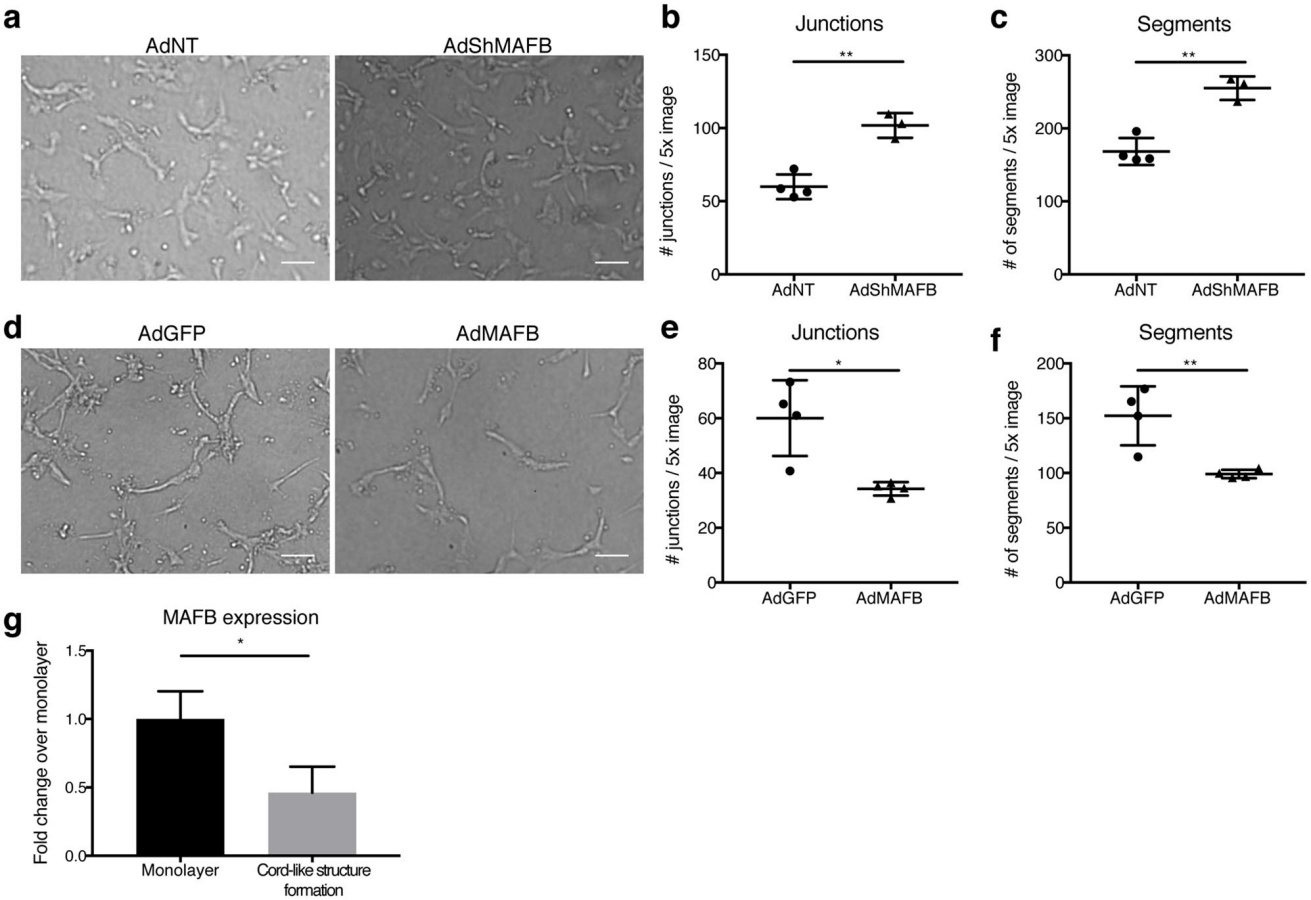
MAFB controls tubular morphogenesis of cultured LECs. **a** Representative images of cord morphogenesis of LECs transduced with an adenoviral vector to downregulate MAFB (AdShMAFB) and a control vector (AdNT). Quantification of the number of junctions (**b**) and cord segments (**c**). **d** Representative images of cord morphogenesis by LECs transduced with a MAFB overexpression vector (AdMAFB) in comparison to a control vector (AdGFP). Quantification of junctions (**e**) and segments (**f**). **g** LECs were seeded and left untreated or subjected to cord morphogenesis. After 16 h, cells were lysed and MAFB expression was quantified by qPCR and expressed as fold change compared to untreated cells (steady state). Scale bars: 100 μm. Data represent mean ± SD. Significance was determined by unpaired Student’s *t*-test (*n* ≥ 3 replicates/group, representative results of 3 independent experiments), *p < 0.05; **p < 0.01

We previously reported that global deletion of MAFB in mice resulted in a hyper-branching phenotype of the developing dermal lymphatic vasculature at E14.5, characterized by an increase in junction points, whereas the overall vessel length was not affected [10]. In order to determine whether lymphatic branching is controlled by MAFB intrinsically expressed by LECs, we employed an in vitro cord-like structure assay with human dermal LECs transduced with an adenoviral vector to suppress MAFB expression (AdShMAFB) or a corresponding non-targeting control shRNA vector (AdNT) (Fig. S1a). In agreement with our previous in vivo data, MAFB depletion in LECs increased the number of junctions and, correspondingly, of cord segments in this assay (Fig. 1a–c). As the average length per segment was only slightly diminished, this resulted in an increase in the overall length of cord-like structures (Fig. S1b, c). Conversely, overexpression of MAFB in cultured LECs (AdMAFB) (Fig. S1d) significantly decreased the number of junctions and tubular segments (Fig. 1d–f). In this case, the average length per segment significantly increased, resulting in an equal overall length of cord-like structures between AdMAFB and AdGFP-transduced cells (Fig. S1e, f). Of note, neither MAFB knockdown nor overexpression significantly affected proliferation of cultured LECs, indicating that effects on branching were not simply due to changes in cell number (Fig. S1g, h). Furthermore, untransduced LECs downregulated MAFB expression upon initiation of cord-like structure formation, compared to cells grown as a monolayer (Fig. 1g). Together, these data suggest that MAFB is a LEC-intrinsic negative regulator of lymphatic branching.

### Lymphatic MAFB intrinsically controls lymphatic branching during embryonic development

Next, we sought to determine whether LEC-expressed MAFB is indeed responsible for the lymphatic hyper-branching phenotype during embryonic development that we observed previously in global knockout mice [10]. To this end, we generated a conditional, tissue-specific Mafb knockout mouse model by crossing mice with a floxed Mafb locus (Mafb^fl/fl^) [30] with Prox1-CreER^T2^ mice [31], resulting in Mafb deletion in Prox1-expressing cells upon treatment with tamoxifen. To evaluate the efficiency of this model, we treated newborn or adult Prox1-CreER^T2^ × Mafb^fl/fl^ mice with tamoxifen for three or five consecutive days, respectively, and eight or three weeks later, we isolated LECs and blood vessel endothelial cells (BECs) from the ears by FACS sorting (Fig. S2a, b). DNA isolation followed by genomic qPCR using three distinct primer pairs to detect the presence of the Mafb coding sequence demonstrated that the gene was efficiently disrupted in LECs, whereas no recombination occurred in BECs or in Cre-negative Mafb^fl/fl^ littermate controls, using either of the tamoxifen treatment regimens (Fig. S2c–f).

**Fig. 2:**
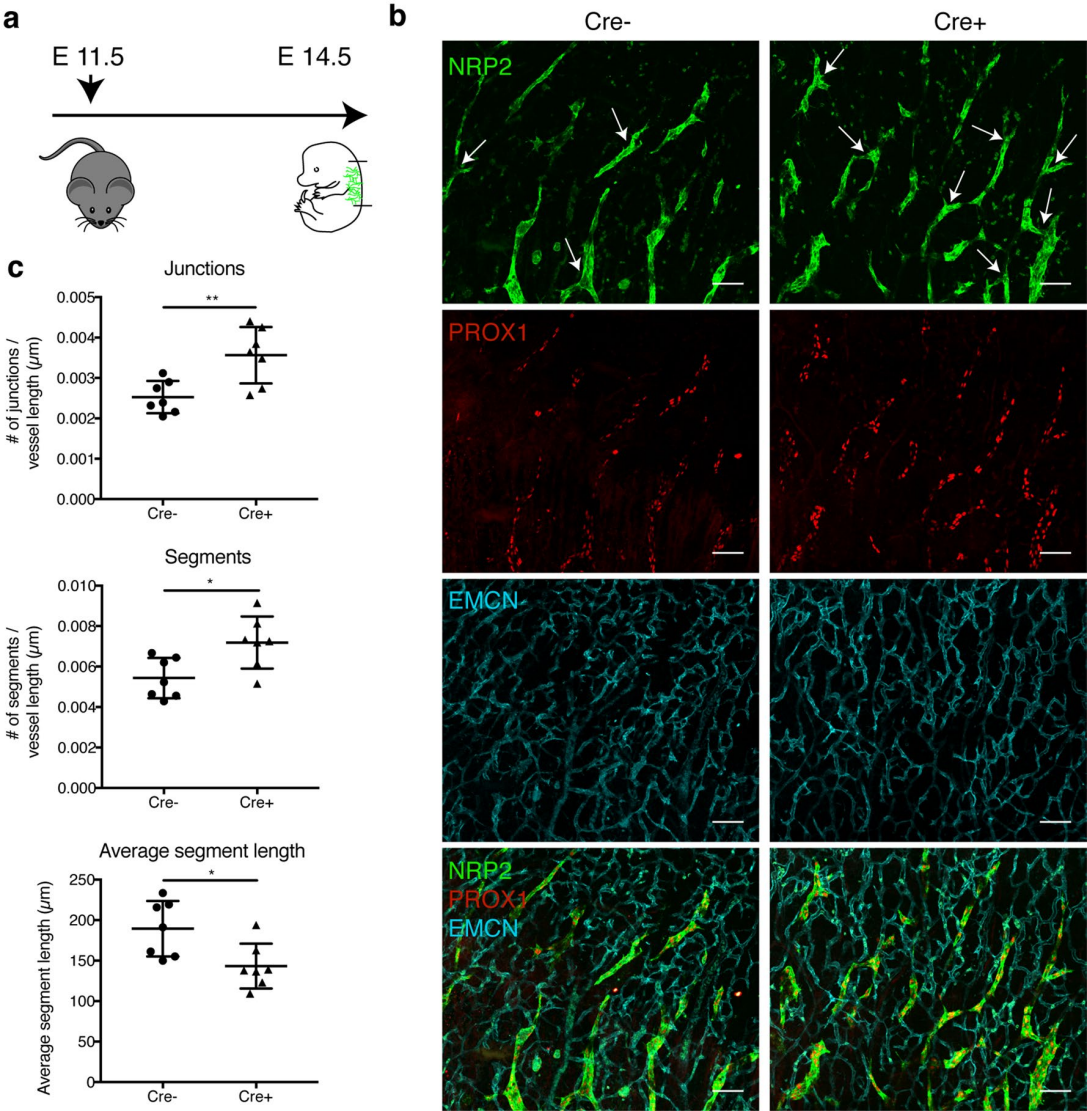
Lymphatic hyper-branching during embryogenesis in E14.5 Prox1-CreER^T2^ × Mafb^fl/fl^ embryos. **a** Schematic of the tamoxifen treatment in pregnant females and analysis of embryos. **b** Representative confocal images (maximum-intensity projections) of E14.5 embryonic back skin stained for NRP2 (green), PROX1 (red), and endomucin (EMCN) (blue). Arrowheads point to vessel junctions (branch points) within the distal sprouting area. **c** Number of vessel junctions and segments normalized to total vessel length and average segment length in the distal sprouting area of Mafb^fl/fl^ (Cre −) and Prox1-CreER^T2^ × Mafb^fl/fl^ (Cre +) littermates (*n* = 7 animals/group). Scale bars: 100 μm. Data represent mean ± SD. Significance was determined by unpaired Student’s t-test, *p < 0.05; **p < 0.01

Next, we used this mouse line to investigate lymphatic development during embryogenesis. Tamoxifen was applied to pregnant females at E11.5, a timepoint at which lymphatic endothelial progenitor cells express Prox1 [11], and lymphatic branching was analyzed in the embryonic back skin at E14.5 (Fig. 2a). Mafb^fl/fl^ littermates served as controls. As expected, MAFB protein was detectable in dermal lymphatic vessels in Mafb^fl/fl^ embryos, but not in Prox1-CreER^T2^ × Mafb^fl/fl^ littermates (Fig S3a). In agreement with our previous findings, we found a significant increase in the number of lymphatic vessel junctions and the number of lymphatic vascular segments (Fig. 2b, c), whereas the average segment length decreased (Fig. 2c). No major effects on the number of filopodia in the tip region of sprouting lymphatic vessels (Fig. S3b, c), on lymphatic vessel area (Fig. S3d) nor on the average size of the LECs (Fig S3e) were observed. Similar to what we reported for global Mafb knockout embryos, depletion of lymphatic MAFB did not alter endomucin (EMCN) + blood vessels (Figs. 2b, S3f). Together, these data clearly demonstrate that the deletion of LEC-expressed MAFB is sufficient to induce hyper-branching in the developing lymphatic system, whereas it has no major effect on the overall growth of lymphatic vessels in the embryonic skin.

### Lymphatic-expressed MAFB regulates lymphatic branching in the diaphragm during postnatal development

**Fig. 3:**
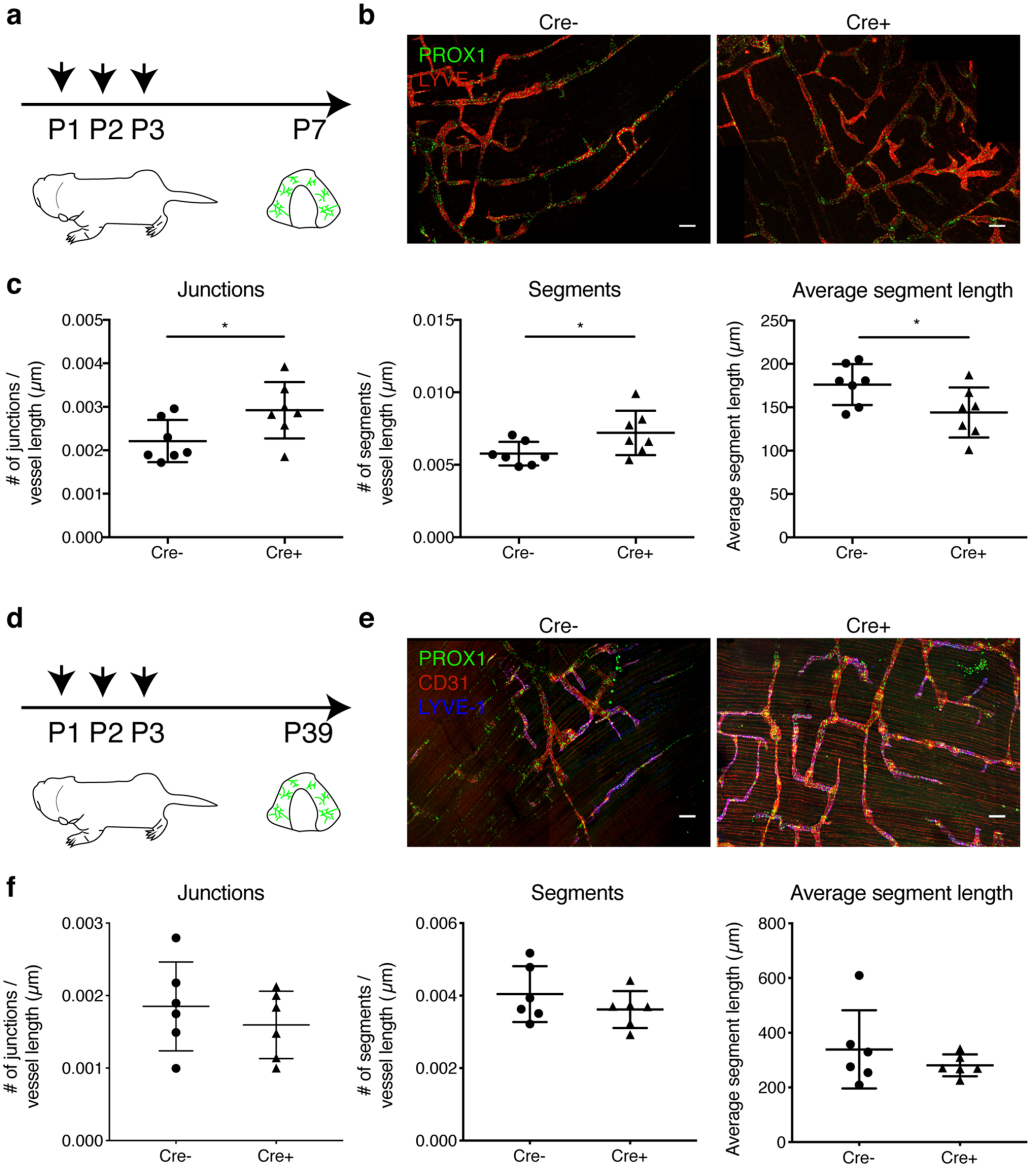
Lymphatic hyper-branching in Prox1-CreER^T2^ × Mafb^fl/fl^ mice during postnatal development. **a** Schematic representation of the tamoxifen treatment schedule and analysis in newborn mice. **b** Representative confocal images (maximum-intensity projections) of P7 diaphragms stained for PROX1 (green) and LYVE-1 (red). **c** Number of vessel junctions and segments normalized to total vessel length and average segment length in the diaphragm (pleural side) of Mafb^fl/fl^ (Cre −) and Prox1-CreER^T2^ × Mafb^fl/fl^ (Cre +) littermates (*n* = 7 animals/group). **d** Schematic representation of the tamoxifen treatment schedule and analysis in adolescent mice. **e** Representative confocal images (maximum-intensity projections) of P39 diaphragms stained for PROX1 (green), CD31 (red), and LYVE-1 (blue). **f** Number of vessel junctions and segments normalized to total vessel length and average segment length in the diaphragm (pleural side) of Mafb^fl/fl^ (Cre −) and Prox1-CreER^T2^ × Mafb^fl/fl^ (Cre +) littermates (*n* = 6 animals/group). Scale bars: 100 μm. Data represent mean ± SD. Significance was determined by unpaired Student’s *t*-test, *p < 0.05

The lymphatic system in the diaphragm undergoes extensive growth and remodeling in the first days after birth, representing a robust and quantifiable system to study postnatal lymphatic development [23]. Thus, we treated Prox1-CreER^T2^ × Mafb^fl/fl^ mice and control Mafb^fl/fl^ littermates with tamoxifen from postnatal day P1 to P3 [32], and analyzed the lymphatic network in the diaphragm at P7 (Fig. 3a). We observed lymphatic hyper-branching, accompanied by an increase in the number of lymphatic vessel segments and a reduction in the average segment length (Fig. 3b, c), while, as in the embryonic back skin, the ratio between Prox1+ LEC nuclei and the lymphatic vascular area was not affected (data not shown). In contrast, we found that neither the development of the mesenteric lymphatic vasculature nor of the lymphatic lacteals in the jejunum was affected in newborn mice lacking lymphatic MAFB (Figs. S4, S5). Thus, lymphatic MAFB does not affect lymphatic collectors in the mesentery or jejunal lacteals which never branch, but restrains branching of capillary lymphatic vessels during active developmental lymphangiogenesis.

### Lymphatic MAFB is dispensable for lymphatic patterning and function in healthy adult mice

**Fig. 4:**
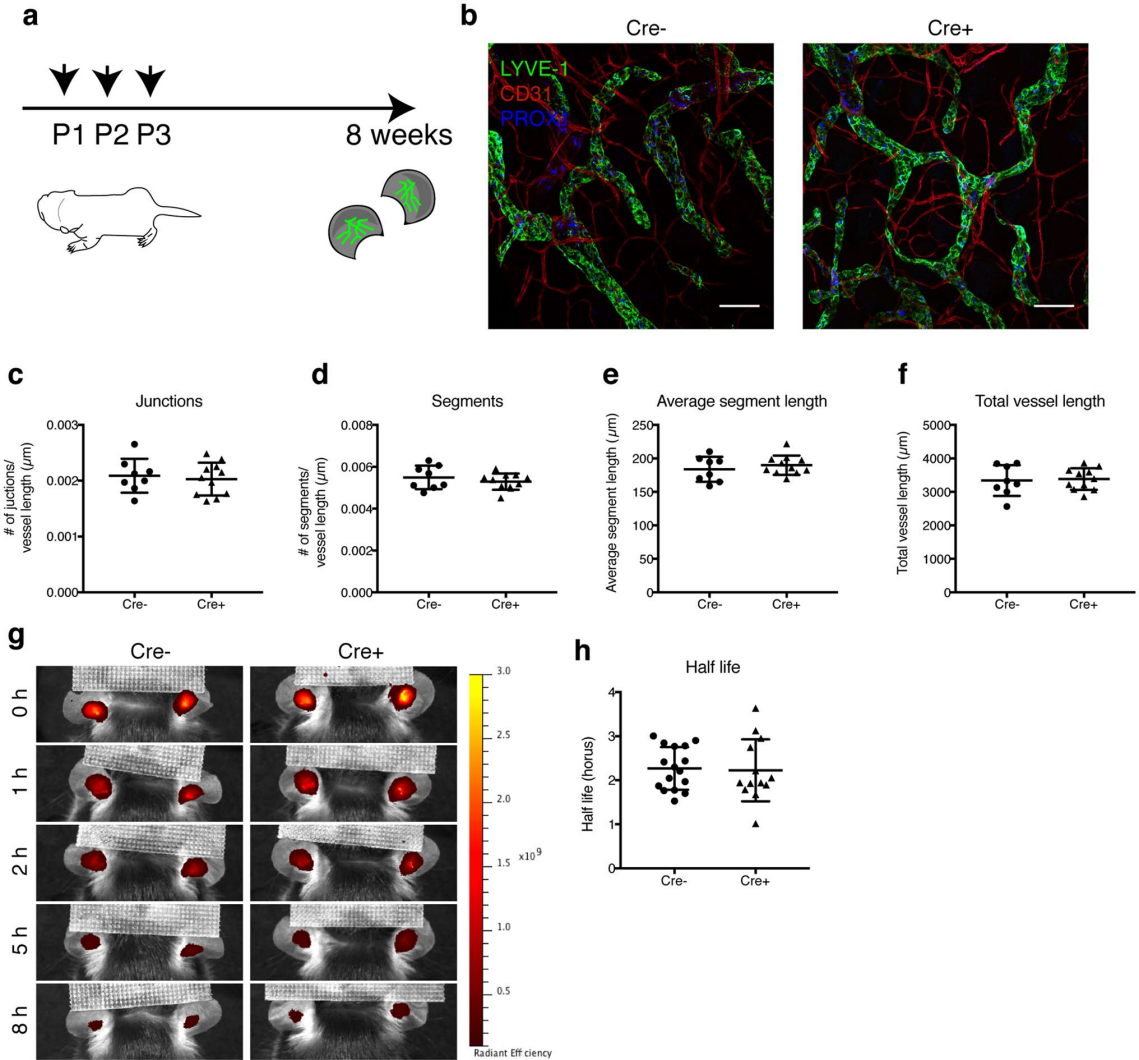
Lymphatic deletion of MAFB is dispensable for dermal lymphatic morphology and drainage capacity in healthy adult mice. **a** Schematic representation of the tamoxifen treatment schedule and analysis in adult mice. **b** Representative confocal images (maximum-intensity projections) of split ear whole mounts of 8-week-old mice stained for LYVE-1 (green), CD31 (red), and PROX1 (blue). Number of vessel junctions normalized to total vessel length (**c**), number of vessel segments normalized to total vessel length (**d**), average segment length, (**e**) and total lymphatic vessel length (**f**) in the ear skin of Mafb^fl/fl^ (Cre −) and Prox1-CreER^T2^ × Mafb^fl/fl^ (Cre +) mice (n ≥ 8 animals/group). Scale bar: 100 μm. **g** Representative images showing clearance of an intradermally injected near-infrared lymphatic tracer (PEG20-IRDye800) in ears of Mafb^fl/fl^ (Cre −) and Prox1-CreER^T2^ × Mafb^fl/fl^ (Cre +) mice. **h** Quantification of tracer half-life in the ear skin (*n* ≥ 13 animals/group). Data represent mean ± SD. Significance was determined by unpaired Student’s t-test

Since deletion of lymphatic MAFB resulted in an altered lymphatic patterning and hyper-branching during development, we wondered whether these defects would translate into structural or functional impairments in adult mice. To investigate this question, we treated newborn Prox1-CreER^T2^ × Mafb^fl/fl^ mice and control Mafb^fl/fl^ littermates with tamoxifen from P1 to P3, and analyzed the lymphatic morphology in the diaphragm once the mice reached adolescence (Fig. 3d). Interestingly, the hyper-branching phenotype seen in newborn pups was completely rescued in adolescent mice (Fig. 3e, f). Similarly, no major changes in the lymphatic density or branching were observed in the ear skin of 8 weeks old mice (Fig. 4a–f). Overall, our results indicate that defects induced by a lack of MAFB in developing lymphatic networks are only transient and can be compensated during final maturation.

As a major function of the lymphatic system is to provide fluid drainage of peripheral tissues, we also assessed the transport capacity of dermal lymphatics in adult Prox1-CreER^T2^ × Mafb^fl/fl^ mice and control Mafb^fl/fl^ littermates treated with tamoxifen. To do so, we injected a near-infrared lymphatic tracer (PEG20-IRDye800) into the ear dermis [33], and monitored its fluorescence decay (corresponding to the lymphatic clearance of the tracer) over time. In agreement with the overall normal lymphatic morphology, no difference in tracer clearance was detected between Prox1-CreER^T2^ × Mafb^fl/fl^ mice and control Mafb^fl/fl^ mice (Fig. 4g, h), indicating that MAFB is dispensable for the lymphatic structure and function in healthy adult mouse skin.

### Lymphatic MAFB restricts pathological lymphangiogenesis in adult mice

**Fig. 5:**
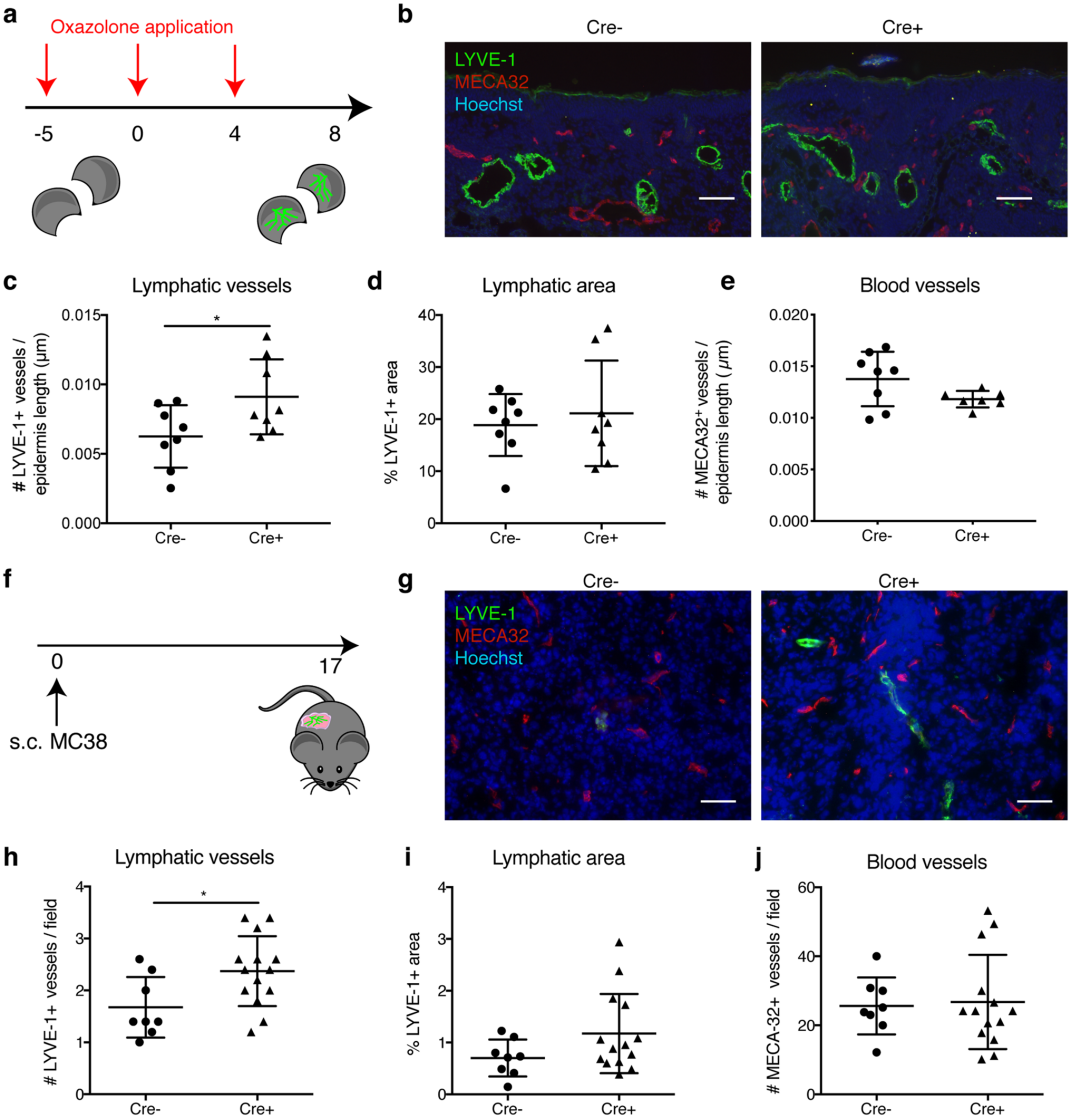
Lymphatic MAFB deletion increases tumor-associated lymphangiogenesis. **a** Schematic representation of the treatment schedule with oxazolone to induce cutaneous hypersensitivity reactions in the ear skin. Mice were sensitized on day −5 and challenged by oxazolone application to the ears on days 0 and 4. **b** Representative immunofluorescence images of ear sections from Mafb^fl/fl^ (Cre −) and Prox1-CreER^T2^ × Mafb^fl/fl^ (Cre +) mice stained for LYVE-1 (green), MECA-32 (red), and Hoechst (blue). Scale bars: 100 μm. Quantification of the lymphatic vessel number (normalized to epidermis length, *n* = 8 animals/group) (**c**), percentage of the LYVE-1 stained area (*n* = 8 animals/group) (**d**), and the number of MECA-32 + blood vessels (normalized to epidermis length, *n* = 8 animals/group) (**e**). **f** Schematic representation of the subcutaneous MC38 tumor model. **g** Representative immunofluorescence images of MC38 tumors from Mafb^fl/fl^ (Cre −) and Prox1-CreER^T2^ × Mafb^fl/fl^ (Cre +) mice stained for LYVE-1 (green), MECA-32 (red), and Hoechst (blue). Scale bars: 50 μm. Quantification of the lymphatic vessel number per field (*n* ≥ 8 animals/group) (**h**), percentage of the LYVE-1 stained area (*n* ≥ 8 animals/group) (**i**), and the number of MECA-32 + blood vessels per field (*n* ≥ 8 animals/group) (**j**). Data represent mean ± SD. Significance was determined by unpaired Student’s *t*-test, *p < 0.05

The function of lymphatic MAFB in regulating vessel branching might also be apparent during other phases of active lymphangiogenesis. Since the lymphatic network is largely stable and does not undergo major remodeling in healthy adults, we decided to pathologically trigger neo-lymphangiogenesis in adult Prox1-CreER^T2^ × Mafb^fl/fl^ mice and control Mafb^fl/fl^ littermates. We applied tamoxifen to young adults (6–7 weeks old) for five consecutive days. Then, we used a well-established oxazolone sensitization and challenge protocol (Fig. 5a) to elicit a cutaneous hypersensitivity response in the ear skin which is accompanied by extensive lymphatic remodeling [27, 28, 34]. Deletion of lymphatic MAFB did not affect ear inflammation (Fig. S6a) and also had no major effect on the number of lymphatic vessels in the inflamed ears. While there was a slight increase in the number of lymphatic vessel profiles in tissue sections of Prox1-CreER^T2^ × Mafb^fl/fl^ mice stained for LYVE-1 (Fig. 5b, c), the LYVE-1+ area and average lymphatic vessel size were identical (Figs. 5d and S6b). Tissue wholemount stainings furthermore showed largely comparable lymphatic vessel morphology and branching (Fig. S6c–f). The number and area of blood vessels was not affected either (Figs. 5e and S6g), nor was the lymphatic drainage function (Fig. S6h, i).

A setting in which pathological lymphangiogenesis and remodeling is more strongly induced is during tumor growth [25, 26]. Thus, we subcutaneously implanted BL/6 syngeneic MC38 colorectal carcinoma cells which express high levels of VEGF-C [35], allowed the tumors to develop for 17 days, and then assessed lymphatic vessels within the tumors histologically (Fig. 5f). Deletion of lymphatic MAFB increased the number of intra-tumoral lymphatic vessels (Fig. 5g, h), but had no significant effect on the overall area covered by lymphatic vessels (Fig. 5i), nor on the formation of tumor-associated blood vessels (Fig. 5j). The absence of MAFB from lymphatic vessels did not change the weight of tumor-draining lymph nodes (Fig. S6j) or the abundance of migratory dendritic cells arriving in the nodes (Fig. S6k), suggesting that lymphatic drainage of the tumor bed is similar in Cre− and Cre+ animals.

Taken together, our data reveal that lymphatic MAFB regulates lymphatic branching and morphogenesis during active developmental and pathological lymphangiogenesis, but is not required for the normal lymphatic architecture and function in healthy adult mice.

## Discussion

While the initial steps leading to the embryonic development of the lymphatic system have been described to considerable extent [14], the precise signaling events and transcriptional programs that lead to LEC specification and differentiation and that control the formation and maturation of the lymphatic system in the growing organism are still not completely understood. VEGF-C signaling plays an important role in these processes, not only by guiding the sprouting and migration of LECs from the cardinal vein [17], but also by regulating the expression of PROX1 [18, 36]. Recently, the transcription factor HHEX was shown to be a critical upstream regulator of the VEGF-C/VEGFR-3 axis during early lymphatic development, and its absence in the vasculature resulted in severe lymphatic defects during embryogenesis [37]. Furthermore, specific signal transduction events, including VEGFR-3 phosphorylation and subsequent induction of ERK, have been identified in the developing lymphatic system [38]. However, the transcriptional events *downstream* of VEGFR-3 activation that are responsible for its developmental role are still largely unknown.

We and others have previously identified the transcription factor MAFB as a direct target of VEGF-C/VEGFR-3 signaling in LECs [9, 10]. Functionally, we found that global MAFB deletion in mice resulted in an early lymphatic patterning defect, manifesting in a hyper-branched lymphatic network in the skin of E14.5 embryos which in terms of overall vessel growth, however, appeared relatively normal [10]. In line with this, zebrafish carrying a mutant, non-functional MAFB primarily had deficits in LEC migration, but not in VEGF-C induced LEC proliferation [9]. Of note, in both these studies, MAFB was deleted or mutated globally. Thus, it has remained unclear whether MAFB intrinsically expressed by LECs is required for normal lymphatic development, or whether MAFB expressed by other cell types, in particular macrophages, is involved. To investigate these issues, we established a mouse model with conditional deletion of MAFB specifically in LECs. We chose the Prox1-CreER^T2^ driver mouse line as it currently is the most lymphatic-specific Cre mouse line that spares the myloid/macrophage compartment, in contrast to e.g., the LYVE-1-Cre mouse. Using this model, we show that LEC-specific deletion of MAFB fully recapitulates the embryonic lymphatic defects observed previously in global Mafb knockout mice, i.e., a hyper-branching of the dermal lymphatic network. In contrast to global MAFB deletion which is perinatally lethal [22], lymphatic-specific MAFB deletion also allowed us to study postnatal lymphatic development in the absence of lymphatic MAFB. Interestingly, lymphatic hyper-branching was also evident in the capillary network of the diaphragm at P7 and after pathologically induced neo-lymphangiogenesis in tumor-bearing adult mice. On the other hand, vessels that do not branch such as the mesenteric collectors and lacteals were not affected.

The lymphatic hyper-branching phenotype after global or tissue-specific MAFB deletion was subtle, and did not affect the lymphatic network morphology or the lymphatic transport function in healthy adult mice. Possibly, functional redundancy between MAFB and the other large MAF transcription factors (MAFA and c-MAF) might be a reason why we did not observe more striking effects on the lymphatic system. Similarly, single MAFB deletion had only very subtle effects on the differentiation of e.g., macrophages [3, 39], although MAFB is highly expressed in these cells. In contrast, double-knockout of MAFB and c-MAF clearly altered the proliferative potential of myeloid cells [39], suggesting that large MAF transcription factors can indeed compensate for each other, at least to some extent.

Currently, the regulation of lymphatic branching is poorly understood. Apart from VEGF-C, other extrinsic factors controlling lymphatic sprouting and branching have been identified, such as TGF-ß [40] and Wnt5a [41]. Further studies are required to investigate whether MAFB is also activated by any of those factors. Furthermore, the transcriptional targets of MAFB that mediate its function during the sprouting process in LECs still need to be identified. We previously found that MAFB regulates several LEC differentiation genes, including Prox1 and VEGFR-3, but their involvement in vascular branching is not fully understood. Possibly, incomplete or altered differentiation of LECs due to MAFB deletion might delay the maturation and pruning of the growing lymphatic network, resulting in a transient relative increase of network complexity with more junctions and vessel branches. Another potential explanation for the hyper-branching phenotype could be that MAFB regulates the cytoskeleton and cellular morphology of LECs. In this regard, a previous report described that MAFB deletion in macrophages resulted in increased formation of branched, cellular protrusions in response to stimulation, due to alterations in the actin cytoskeleton [39]. However, we found no difference in the formation of filopodia in growing dermal lymphatic sprouts at E14.5 after MAFB deletion. Furthermore, MAFB has also been implicated in LEC migration in zebrafish, so it is tempting to speculate that MAFB may control lymphatic vascular branching by regulating LEC migration (and, potentially, anastomosis of nearby sprouts) in response to VEGF-C gradients. Interestingly, MAFB appears to exert a different function during blood vessel development, as it promoted retinal vessel sprouting [42]. Thus, the activation and or the transcriptional targets of MAFB likely differ in blood and lymphatic vessels, but additional studies are needed to pinpoint the molecular basis for these divergent roles of MAFB in different types of vascular beds.

## Material and methods

### Cell culture, adenoviral transduction, cord formation, and proliferation assay

Primary human dermal microvascular LECs [43] were cultured under standard culture conditions (37 °C and 5% CO2) on collagen-I (Advanced BioMatrix) coated dishes (50 µg/mL) in EBM medium (Lonza) containing 20% FBS (Gibco), 1% penicillin/streptomycin (Gibco), 2 mmol/L l-glutamine (Gibco), 25 µmol/mL cAMP (Sigma-Aldrich) and 10 µg/mL hydrocortisone (Sigma-Aldrich). For overexpression of MAFB, ready-made adenoviral vectors (Sirion Biotech) with the human MAFB cDNA under control of the cytomegalovirus (CMV) promoter (AdMAFB) were used. A CMV:GFP (AdGFP) vector served as a control. For silencing of MAFB, a ready-made adenoviral vector (Sirion Biotech) with a pre-validated shRNA targeting MAFB under the U6 promoter (AdShMAFB) and a non-targeting control shRNA construct (AdNT) were used. For transduction, LECs were infected with adenovirus at a MOI of 25 for AdGFP and AdMAFB or at a MOI of 50 for AdNT and AdShMAFB. Four hours after the infection, the medium was replaced and cells were subjected to a cord-like structure morphogenesis assay essentially as described previously [20]. In brief, LECs were seeded on collagen-coated 24-well plates (8 × 10^4^/well) and grown to confluency. Cells were incubated for 8 h in EBM plus 5% FBS and subsequently overlaid with 500 µL of a collagen hydrogel (1 mg/mL). Endothelial cord-like structures were imaged 16 h later using an inverted microscope (Zeiss) and analyzed using ImageJ. RNA was isolated from cells cultured as monolayers or after formation of cord-like structures, 72 h after adenoviral infection, using the Nucleospin RNA kit (Macherey–Nagel) according to the manufacturer’s instructions. RNA concentrations were measured using a NanoDrop ND-1000 spectrophotometer (Witec), and retrotranscribed using the High-Capacity cDNA Reverse Transcription Kit (Applied Biosystems).

To assess proliferation, adenovirus-transduced LECs were seeded in collagen-coated 96-well plates (4000/well) and cultured for the indicated time periods. Proliferation was analyzed by adding 100 µg/mL 4-methylumbelliferyl heptanoate (MUH, Sigma-Aldrich) in PBS and measurement on a SpectraMax Reader (Molecular Devices) at 355 nm excitation and 460 nm emission [44].

### Mice

To create the conditional lymphatic-specific MAFB knockout mouse model, mice carrying a Mafb gene flanked by loxP elements [30] were crossed with Prox1-CreER^T2^ mice [31], kindly provided by Dr. Taija Mäkinen, Uppsala University, Sweden) to generate Prox1-CreER^T2^ × Mafb^fl/fl^ mice on the C57BL/6 background. To induce embryonic deletion of MAFB, pregnant females were injected intraperitoneally at E11.5 with 50 mg/kg of tamoxifen (Sigma) dissolved in ethanol/sunflower seed oil. To induce postnatal gene deletion, tamoxifen was administered intragastrically (50 µg per dose) to pups daily from P1 to P3 [32]. In another set of experiments, adult mice were administered tamoxifen intraperitoneally (50 mg/kg) for 5 consecutive days and analyzed 3 weeks after the last injection. Prox1-CreER^T2^ × Mafb^fl/fl^ and Mafb^fl/fl^ control littermates were subjected to the same treatment regimen and all the experiments were performed in a blinded fashion. All mice used in this study were bred and housed in an SOPF animal facility of ETH Zurich and experiments were performed in accordance with animal protocols approved by the local veterinary authorities (Kantonales Veterinäramt Zürich).

### LEC isolation and determination of recombination efficiency

For isolation of primary mouse endothelial cells, ears of Prox1-CreER^T2^ × Mafb^fl/fl^ and Mafb^fl/fl^ tamoxifen treated mice were split, minced, and digested with collagenase IV (Life Technologies) and DNaseI (Roche) for 30 min at 37 °C under constant agitation. Samples were passed through a 40 µm cell strainer, washed twice with FACS buffer (PBS, 1% FBS, 2 mM EDTA), and stained with primary antibodies [rat anti-mouse-CD45 antibody conjugated to APC–Cy7 (BioLegend 103115, 1:200); rat anti-mouse-CD31 antibody conjugated to APC (BD 551262, 1:300); hamster anti-mouse-podoplanin antibody conjugated to PE (eBioscience 12-5381, 1:400)] for 20 min on ice. 7AAD was used for discrimination of living and dead cells. Sorting of 7AAD – CD45 – CD31 + podoplanin + singlet LECs and 7AAD – CD45 – CD31 + podoplanin – singlet BECs was done using a FACS Aria II instrument (BD). DNA from LECs and BECs was isolated using the Nucleospin Tissue XS kit (Macherey–Nagel) according to the manufacturer’s instructions.

### Quantitative polymerase chain reaction

Gene expression in human LECs as well as MAFB knockout efficiency in mouse LECs and BECs was measured by qPCR using the PowerUp SYBR green master mix (Thermo Fisher) on a QuantStudio 7 Flex system or an 7900 HT Fast instrument (Applied Biosystems). GAPDH served as an internal control. Relative expression of genes was calculated according to the 2^−ΔCT^ formula. Primer sequences for human cells were: MAFB-fwd: TCAAGTTCGACGTGAAGAAGG; MAFB-rev: GTTCATCTGCTGGTAGTTGCT; GAPDH-fwd: 5′-CATGAGAAGTATGACAACAGC-3′; GAPDH-rev: 5′-AGTCCTTCCACGATACCAAAG-3′. Primer sequences for isolated mouse cells were: MAFB1-fwd: TTCGACGTGAAGAAGGAGCC; MAFB1-rev: GTAGTTGCTCGCCATCCAGT; MAFB2-fwd: TGAGCATGGGGCAAGAGCTG; MAFB2-rev: CCATCCAGTACAGGTCCTCG; MAFB3-fwd: AGGGTATGACTGTGTGTGCT; MAFB3-rev: CAAGCCAGAATGCAAAAGCG; GAPDH-fwd: CCTGGAGAAACCTGCCAAGTATG; GAPDH-rev: AGAGTGGGAGTTGCTGTTGAAGTC.

### Morphological analysis of lymphatic vessels in tissue whole mounts

Back skin from E14.5 embryos and diaphragms, small intestine and mesenteries from P7 pups or P39 adolescent mice were collected. Tissue wholemounts were fixed with paraformaldehyde (PFA), blocked in blocking solution (5% donkey serum, 0.2% BSA, 0.3% Triton X-100, and 0.05% NaN3 in PBS), and stained with primary antibodies (in blocking solution) O/N at 4 °C, followed by washing in PBS and incubation with secondary antibodies in PBS for 2 h at RT. Primary antibodies were: rat anti-CD31 (1:200, BD Biosciences, 550274), rabbit anti-LYVE-1 (1:600, AngioBio, 11-034), rabbit anti-PROX1 (1:200, Angiobio, 11-002), goat anti-PROX1 (1:200, R&D, AF2727), goat anti-NRP2 (1:200, R&D, AF567), rat anti-endomucin (1:200, SantaCruz, sc-53941) and rabbit anti-MAFB (1:200, Sigma, HPA005653). Secondary antibodies were: donkey anti-goat AlexaFluor488, donkey anti-rat AlexaFluor488, donkey anti-rabbit AlexaFluor488, donkey anti-rabbit AlexaFluor594, donkey anti-goat AlexaFluor594, donkey anti-rat AlexaFluor647, and donkey anti-rabbit AlexaFluor647 (all from Life Technologies). Confocal images (z-stacks) were taken with an LSM780 or LSM880 microscope (Zeiss), and maximum-intensity projections and image analysis were done with ImageJ (NIH). Network parameters (number of junctions, number of segments, average segment length) were determined after manually marking each vessel using the “Analyze skeleton” plugin.

### Lymphangiogenesis determination in tissue sections

OCT-embedded tissue samples from Prox1-CreER^T2^ × Mafb^fl/fl^ and Mafb^fl/fl^ mice were frozen with liquid nitrogen, and 7-μm cryostat sections were prepared. After fixation in acetone and rehydration in 80% methanol, the sections were blocked (5% donkey serum, 0.2% BSA, 0.3% Triton X-100, and 0.05% NaN3 in PBS), followed by incubation with the respective primary antibodies [goat anti-mouse LYVE-1 (1:100, R&D, AF2125) and rat anti-mouse MECA-32 (1:200, BD Biosciences, 553849)] O/N at 4 °C. After washing, sections were incubated with donkey anti-goat AlexaFluor488 and donkey anti-rat AlexaFluor594 secondary antibodies together with Hoechst 33342 (all from Life Technologies) for 1 h at room temperature. Slides were mounted with Mowiol mounting medium. Tissue sections were imaged on an Axioskop2 mot plus microscope (Zeiss) with an AxioCam MRc camera (Zeiss). At least 4 images were acquired per sample. For the ears, regions of interest were defined as the area of one ear half between stratum corneum and central cartilage. The LYVE-1 positive area was measured and vessels were counted using ImageJ in a blinded fashion. Results are expressed as positive area, vessel count and vessel size. For the ears, the number of vessels was normalized to the basement membrane length, as inflammatory edema causes an increase in tissue area.

### Lymphatic clearance assay

The polyethylene glycol-conjugated lymphatic tracer PEG20-IRDye800 was prepared as described previously [33]. To examine lymphatic clearance over time, healthy or oxazolone inflamed mice (8 days after oxazolone challenge) were anesthetized with isoflurane, and 3 μL of 3 μM tracer was injected intradermally into the ear skin [45]. The mice were positioned in an IVIS Spectrum imaging system and an image was acquired just after tracer injection, with an exposure of 2 s (λex: 745 nm, λem: 800 nm, binning of 4), and then at 1 h, 2 h, 5 h and 8 h after the injection. Between the different imaging timepoints, mice were allowed to wake up and move freely. In order to calculate tissue enhancement values, all signal intensities were adjusted to baseline ear signals before tracer injection. The tissue enhancement value obtained directly after the injection of the tracer was used to normalize all values of the subsequent measurements. A 1-phase exponential decay model was used [45] to fit a decay curve for each mouse, from which the lymphatic clearance expressed as tracer half-life was deduced (Half-life = ln(2/*K*), where ln is the natural logarithm and *K* is the decay constant).

### Cutaneous hypersensitivity assay

A CHS response was induced in the ear skin of 8-week-old, tamoxifen treated Prox1-CreER^T2^ × Mafb^fl/fl^ and Mafb^fl/fl^ mice as described [46]. Briefly, mice were anesthetized by isoflurane inhalation and sensitized by topical application of 2% oxazolone (4-ethoxymethylene-2 phenyl-2-oxazoline-5-one; Sigma) in acetone/olive oil (4:1 vol/vol) on the shaved abdomen (50 μL) and on each paw (5 μL). Five days after sensitization, 10 μL of a 1% oxazolone solution were applied topically to each side of the ears and again 4 days later. The ear thickness was measured every other day until the end of the experiment using a caliper. The increase in ear thickness over baseline levels was used to measure the extent of inflammation.

### MC38 tumor model

Eight-week-old female Prox1-CreER^T2^ × Mafb^fl/fl^ and Mafb^fl/fl^ mice were treated with tamoxifen (50 mg/kg) for 5 consecutive days. Three days after the last tamoxifen injection, mice were shaved and injected with 1 × 10^5^ MC38 cells (kindly provided by Dr. Tiziana Schioppa, Humanitas Clinical and Research Center, Milan, Italy) in 50 µL PBS subcutaneously in the flank and tumors were grown for 17 days. Cells were routinely checked for mycoplasma contamination. At the endpoint, tumors were collected and embedded in OCT for immunofluorescent staining and inguinal and axillary tumor-draining lymph nodes were collected, weighted and processed for flow cytometry. LNs were minced, and digested in LN digestion mix (0.4 mg/mL collagenase IV (Gibco) and 40 μg/mL DNase I (Roche) in DMEM (Gibco) containing 2% FCS and 1.2 mM CaCl_2_ for 30 min at 37 °C. LN fragments were mechanically disaggregated using an automated multichannel pipette before the cell suspension was filtered through a cell strainer. Cells were incubated with Fc blocker (1:50, BioLegend, 101302) prior to staining with fluorescent antibodies for 20 min on ice. Antibodies used were CD45-APC-Cy7 (1:400, BioLegend, 103116), CD11c-PE-Cy7 (1:400, BioLegend, 117318) and MHCII-AF700 (1:800, BioLegend, 107622). Live/dead cell staining with Zombie-Aqua (1:500, BioLegend, 423102) was done together with the antibody incubation. After washing, cells were resuspended in FACS buffer for acquisition using a Beckman Coulter CytoFLEX S flow cytometer. FACS data were analyzed using FlowJo version 10.5.3 software (BD Biosciences).

### Statistical analyses

Statistical analyses were performed using Prism version 7.0a (GraphPad Software Inc.). Data are shown as mean ± SD. To determine statistical significance, a 2-tailed, unpaired Student’s t-test (for the comparison of 2 groups) or a 2-way ANOVA (for grouped analyses and repeated measures) with Bonferroni post-test were performed. Differences were considered statistically significant at *p* < 0.05.

## Electronic supplementary material

Below is the link to the electronic supplementary material.

Supplementary file1 (PDF 2393 kb)
