# Supplementary material for: Lymphatic MAFB regulates vascular patterning during developmental and pathological lymphangiogenesis
Source: Angiogenesis. 2020 Apr 19;23(3):411–23. doi: 10.1007/s10456-020-09721-1 (PMC7311381; doi:10.1007/s10456-020-09721-1)

Lymphatic MAFB regulates vascular patterning during developmental and pathological lymphangiogenesis

Supplementary Figures 1-6

Lothar C. Dieterich[1,*], Carlotta Tacconi[1,*], Franziska Menzi[1], Steven T. Proulx[1], Kübra Kapaklikaya[1], Michito Hamada[2], Satoru Takahashi[2], Michael Detmar[1,$]

[1] Institute of Pharmaceutical Sciences, ETH Zurich, 8093 Zurich, Switzerland

[2] Department of Anatomy and Embryology, Faculty of Medicine, University of Tsukuba, Tsukuba, Ibaraki 305-8575, Japan

[$] Email: michael.detmar@pharma.ethz.ch

* equal contribution

Supplementary Figure 1: (a) MAFB expression in LEC after transduction with an adenoviral vector for MAFB knockdown (AdShMAFB) compared to a vector carrying a non-targeting control shRNA (AdNT) by qPCR. Quantification of average segment length (b) and total length of cord-like structures (c) in AdShMAFB cells compared to control (AdNT) cells. (d) MAFB expression in LECs after transduction with an adenoviral vector for MAFB overexpression (AdMAFB) compared to a control vector (AdGFP) by qPCR. Quantification of average segment length (e) and total length of cord-like structures (f) in AdMAFB compared to control (AdGFP) cells ($n \geq 3$ replicates/group, representative of 3 independent experiments). (g-h) Proliferation of LECs after transduction with AdShMAFB (g), AdMAFB (h), or the corresponding controls, after 24 h, 48 h, and 72 h, determined by MUH fluorescence (pooled data from 4 independent experiments with 4-5 replicates/group each). Significance was determined by unpaired Student's t-test. *$p<0.05$; ****$p<0.0001$.

Supplementary Figure 1

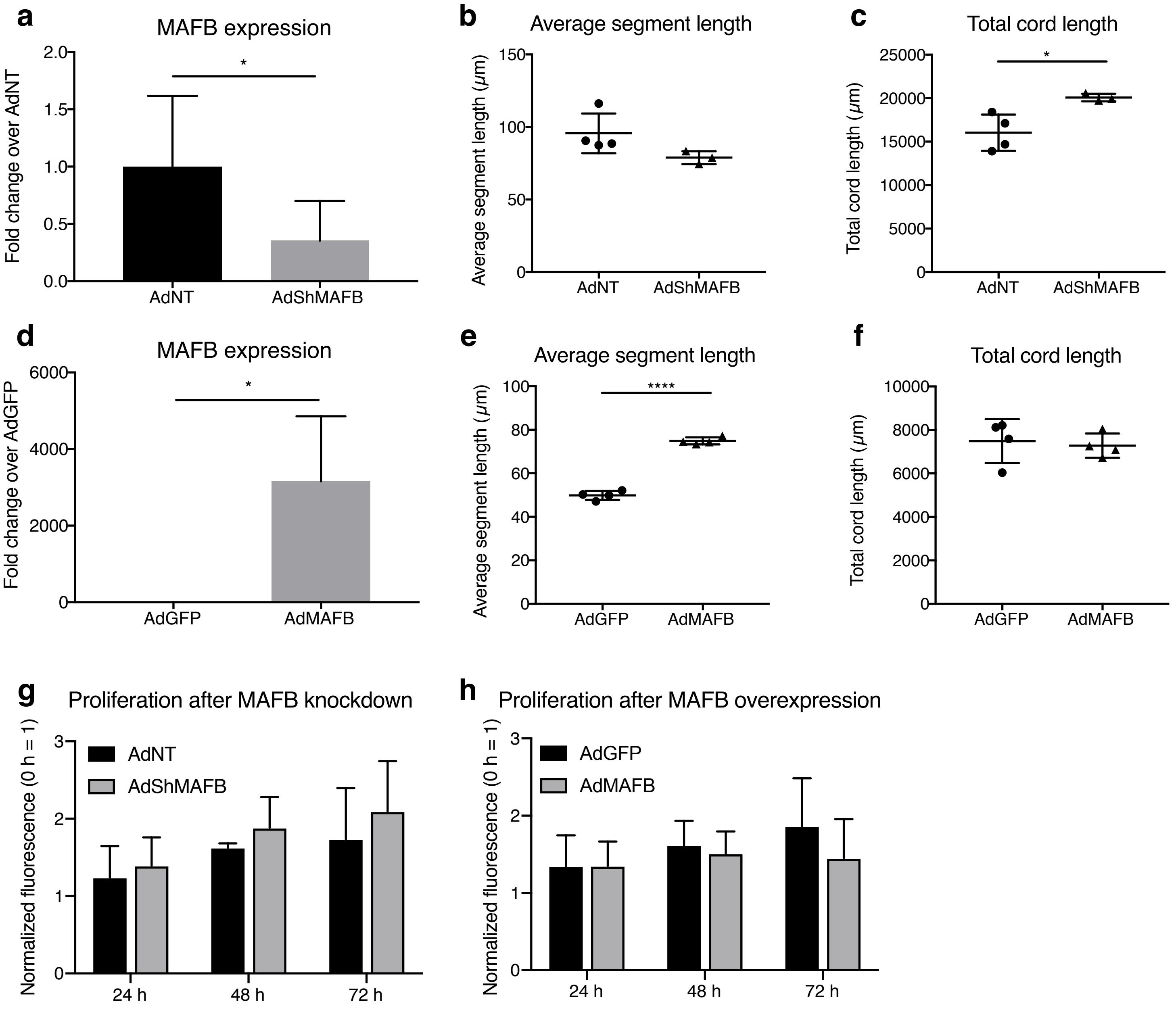

Supplementary Figure 2: (a) Schematic representation of the tamoxifen treatment regimen for newborn pups (upper panel) or adult mice (lower panel) indicated by black arrows and the analysis timepoints. (b) FACS gating strategy used to sort LECs and BECs derived from the ears of age and sex-matched Mafb$^{fl/fl}$ (Cre-) and Prox1-CreER^{T2} x Mafb$^{fl/fl}$ (Cre+) mice. Single, living, CD45- CD31+ cells were separated according to their positivity (LECs) or negativity (BECs) for the lymphatic marker podoplanin (Pdpn). (c) Representation of the mouse Mafb locus showing position of the loxP sites and the primers used in (d), (e) and (f). qPCR analysis of genomic DNA isolated from LECs and BECs obtained from (b) for primer pairs 1 (d), 2 (e) and 3 (f) (n≥4 animlas/group) with tamoxifen treatment in newborn pups (upper panels) or adults (lower panels). Data represent mean ± SD. Significance was determined by unpaired Student's t-test, *$p<0.05$; **$p<0.01$; ***$p<0.001$; ****$p<0.0001$.

Supplementary Figure 2

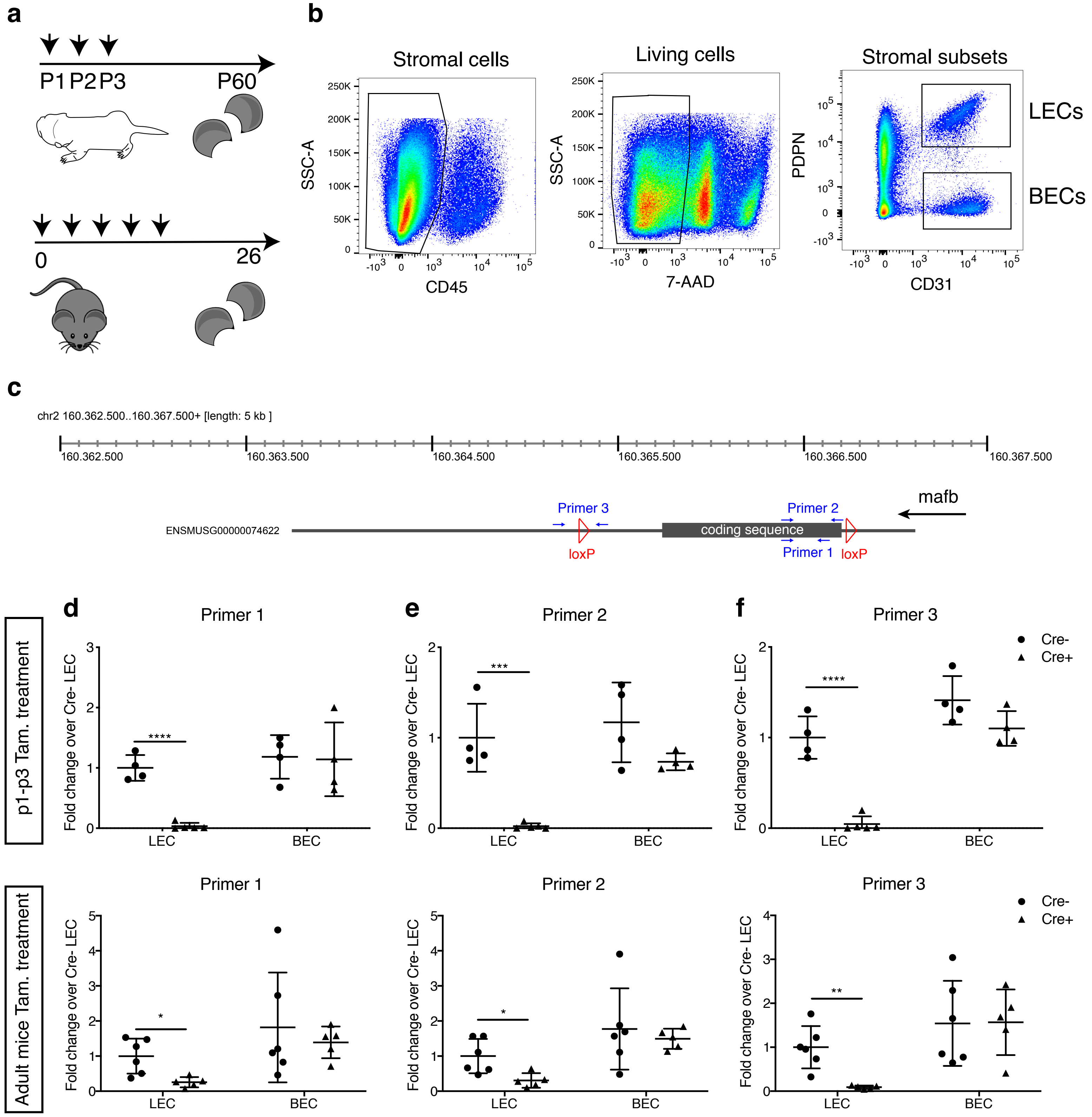

Supplementary Figure 3: (a) Representative confocal images (maximum-intensity projections) of E14.5 embryonic back skin stained for MAFB (cyan) and PROX1 (green) of $Mafb^{fl/fl}$ (Cre-) and Prox1-$CreER^{T2}$ x $Mafb^{fl/fl}$ (Cre+) littermates. Scale bar: 10 μm. White dotted lines indicate the outlines of Prox1-positive LEC nuclei. The white arrow points to a macrophage with strong MAFB expression. (b) Representative confocal images (maximum-intensity projections) of E14.5 embryonic back skin stained for NRP2 (green) and PROX1 (red) of $Mafb^{fl/fl}$ (Cre-) and Prox1-$CreER^{T2}$ x $Mafb^{fl/fl}$ (Cre+) littermates (n=7 animals/group) showing filopodia in the tip region of growing lymphatic vessels. (c) The number of filopodia within the first 50 μm from the tip was counted for $Mafb^{fl/fl}$ (Cre-) and Prox1-$CreER^{T2}$ x $Mafb^{fl/fl}$ (Cre+) littermates (n=7 animals/group). (d) Percentage of the NRP2-stained area (n=7 animals/group) in E14.5 back skin. (e) LEC size (determined by the ratio between the NRP2-positive area and the number of PROX-1-stained lymphatic nuclei) for $Mafb^{fl/fl}$ (Cre-) and Prox1-$CreER^{T2}$ x $Mafb^{fl/fl}$ (Cre+) littermates (n≥6 animals/group). (f) Percentage of the endomucin (EMCN)-stained area (n=7 animals/group). Data represent mean ± SD.

Supplementary Figure 3

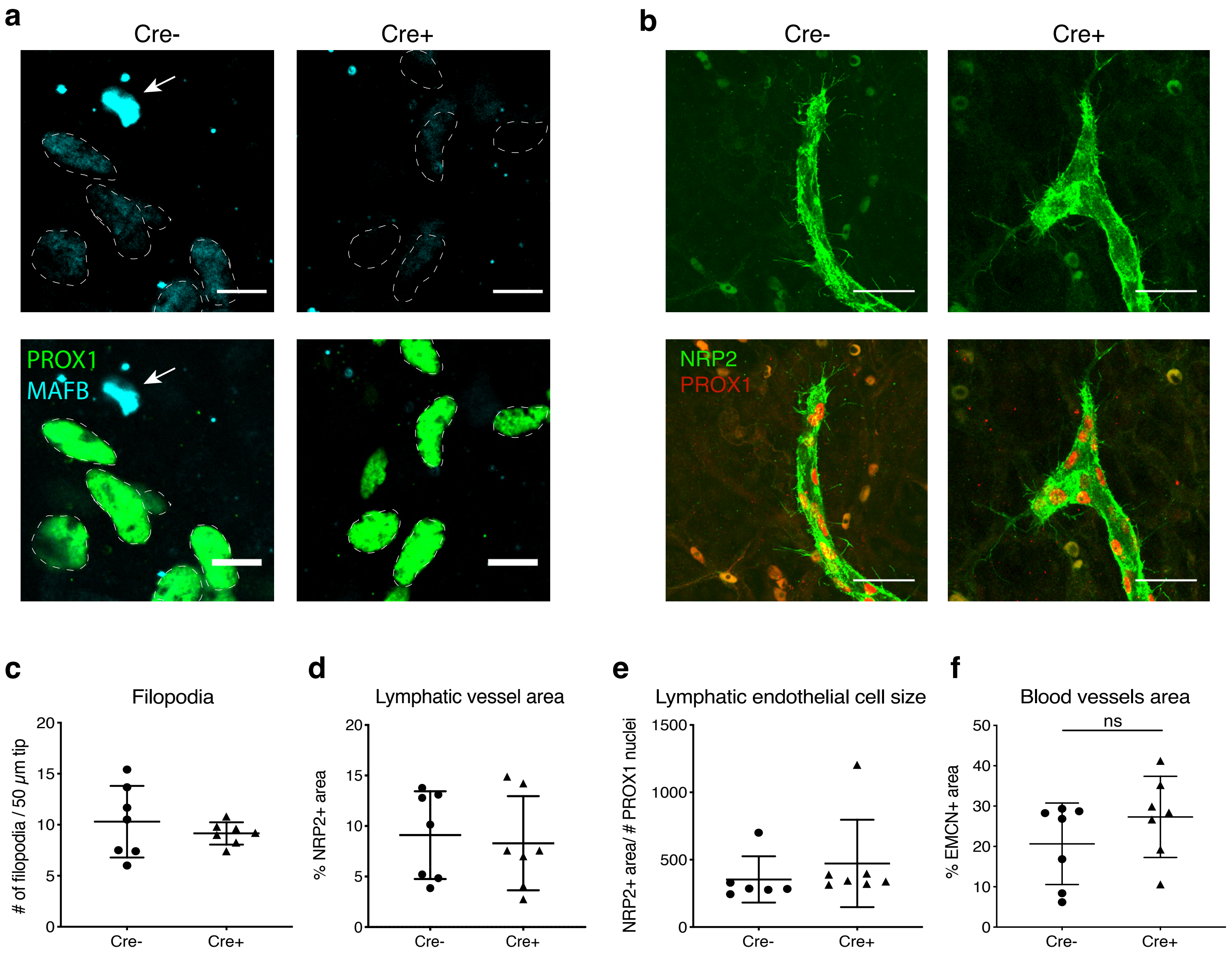

Supplementary Figure 4: (a) Schematic representation of the tamoxifen treatment schedule and analysis in newborn mice. (b) Representative confocal images (maximum-intensity projections) of P7 mesenteries stained for PROX1 (red) and CD31 (green). Number of lymphatic vessel junctions (c) and valves (d) normalized to total vessel length in $Mafb^{fl/fl}$ (Cre-) and Prox1-$CreER^{T2}$ x $Mafb^{fl/fl}$ (Cre+) littermates (n=6 animals/group). Data represent mean ± SD. Scale bar: 100 µm.

Supplementary Figure 4

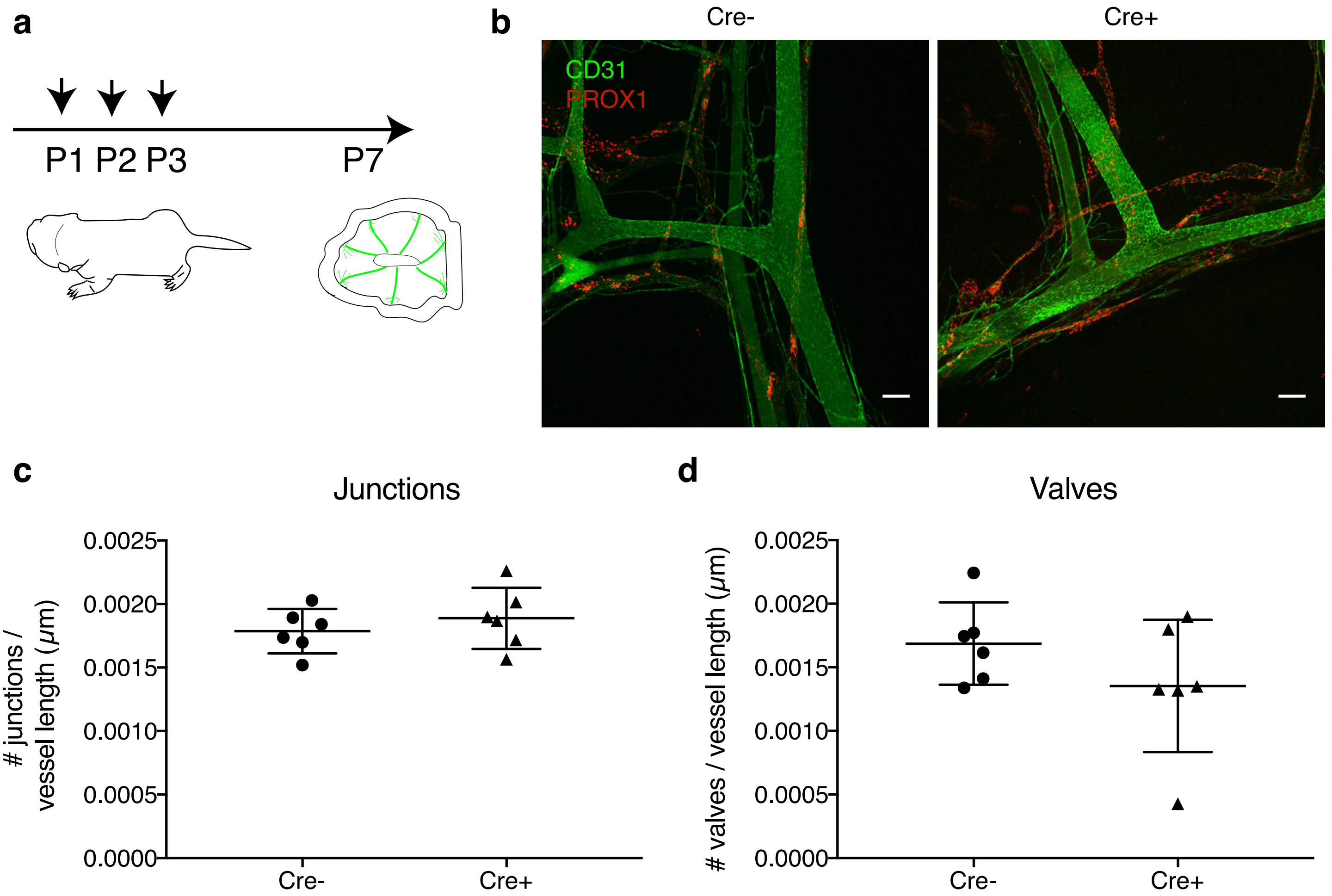

Supplementary Figure 5: (a) Schematic schedule for the treatment with tamoxifen and analysis in newborn mice. (b) Representative confocal images (maximum-intensity projections) of P8 jejunum villi stained for LYVE-1 (green) and CD31 (red). The distance between the tip of the lacteals and the villi (c), the lacteal width (d), and the frequency of lacteals showing filopodia (e) were determined for $Mafb^{fl/fl}$ (Cre-) and Prox1-$CreER^{T2}$ x $Mafb^{fl/fl}$ (Cre+) littermates (n=6 animals/group). Data represent mean ± SD. Scale bar: 100 µm.

Supplementary Figure 5

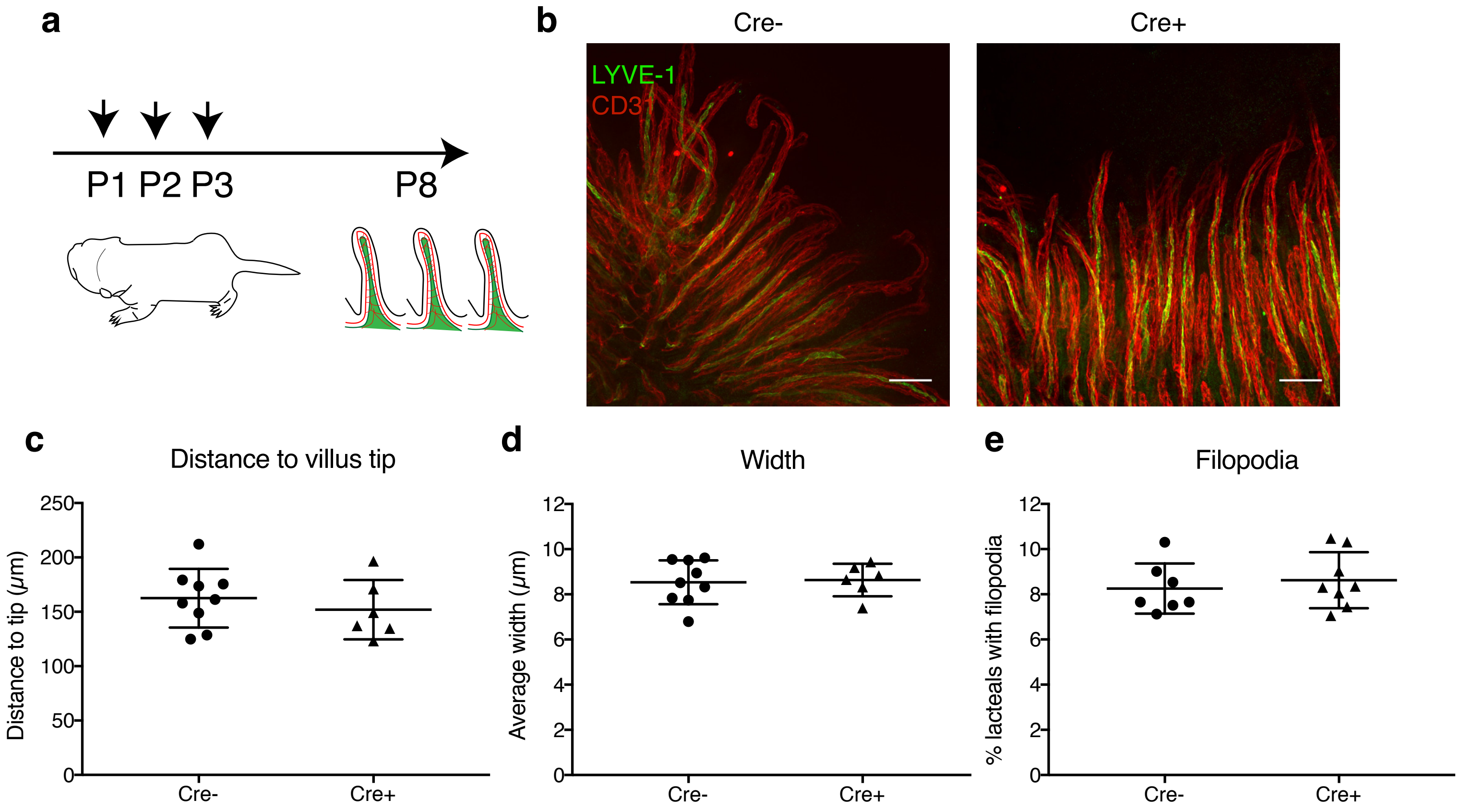

Supplementary Figure 6: (a) Ear thickness represented as change compared to ear thickness prior to challenge with oxazolone of Mafb$^{fl/fl}$ (Cre-) and Prox1-CreER^{T2} x Mafb$^{fl/fl}$ (Cre+) mice (n=8 animals/group). (b) Quantification of the average lymphatic vessel size in inflamed ear sections (n=8 animals/group). (c) Representative confocal images (maximum-intensity projections) of split ear wholemounts of Mafb$^{fl/fl}$ (Cre-) and Prox1-CreER^{T2} x Mafb$^{fl/fl}$ (Cre+) mice subjected to oxazolone induced inflammation stained for LYVE-1 (green) and CD31 (red). (d) Number of vessel junctions normalized to total vessel length, number of vessel segments normalized to total vessel length (e), total lymphatic vessel length (f), and % of CD31+LYVE-1- blood vessel area (g) in the ear skin of Mafb$^{fl/fl}$ (Cre-) and Prox1-CreER^{T2} x Mafb$^{fl/fl}$ (Cre+) mice (n≥4 animals/group). Scale bar: 100 µm. (h) Representative images showing decay of an intradermally injected near-infrared lymphatic tracer (PEG20-IRDye800) in ears of Mafb$^{fl/fl}$ (Cre-) and Prox1-CreER^{T2} x Mafb$^{fl/fl}$ (Cre+) mice on day 7 after the first oxazolone challenge. (i) Quantification of tracer half-life in the inflamed ear skin (n=8 animals/group). (j) Quantification inguinal (Ing) and axillary (Ax) tumor-draining lymph node weight of Mafb$^{fl/fl}$ (Cre-) and Prox1-CreER^{T2} x Mafb$^{fl/fl}$ (Cre+) mice on day 17 after tumor cell injection (n≥10 animals/group). (k) Quantification of migratory dendritic cells (DCs) in tumor-draining lymph nodes defined as single living CD45+ CD11c+ MHCII^{hi} cells of Mafb$^{fl/fl}$ (Cre-) and Prox1-CreER^{T2} x Mafb$^{fl/fl}$ (Cre+) mice on day 17 after tumor cell injection (n≥5 animals/group). Data represent mean ± SD.

Supplementary Figure 6

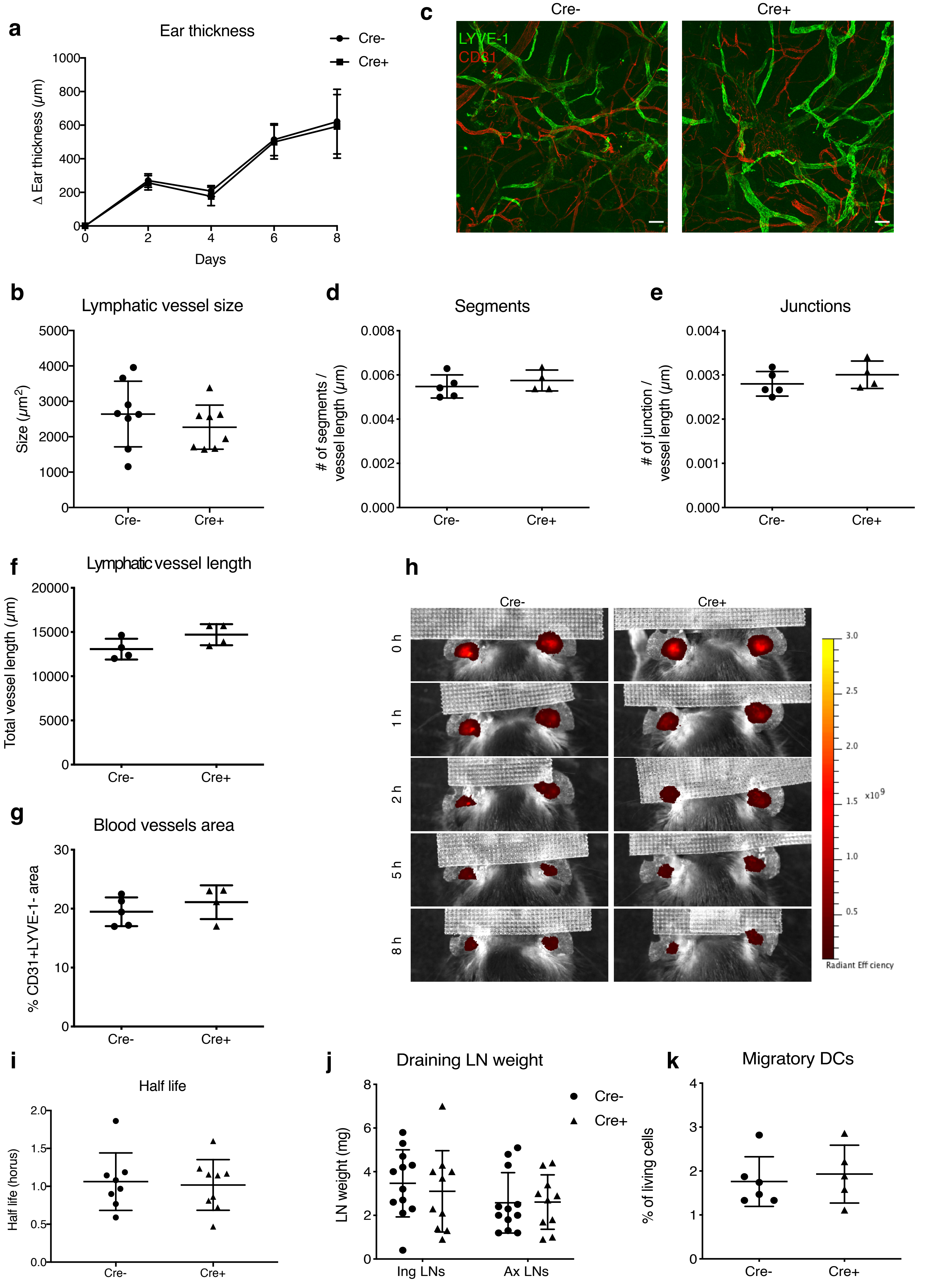

Supplement: Supplementary file 1 — Supplementary file1 (PDF 2393 kb) [file 10456_2020_9721_MOESM1_ESM.pdf]
